# The Use of Extraction on C18-Silica-Modified Magnetic Nanoparticles for the Determination of Ciprofloxacin and Ofloxacin in Meat Tissues

**DOI:** 10.3390/molecules28166123

**Published:** 2023-08-18

**Authors:** Izabella Kośka, Paweł Kubalczyk, Michał Cichomski, Aneta Kisielewska

**Affiliations:** 1 Doctoral School of Exact and Natural Sciences, University of Lodz, Banacha 12/16, 90-237 Lodz, Poland; 2 Department of Environmental Chemistry, Faculty of Chemistry, University of Lodz, Pomorska 163, 90-236 Lodz, Poland; 3 Department of Materials Technology and Chemistry, Faculty of Chemistry, University of Lodz, Pomorska 163, 90-236 Lodz, Poland; michal.cichomski@edu.uni.lodz.pl (M.C.); aneta.kisielewska@edu.uni.lodz.pl (A.K.)

**Keywords:** antibiotics, ciprofloxacin, sorptive extraction, magnetic nanoparticles, ofloxacin

## Abstract

A simple, fast, and low-cost method of extraction using magnetic nanoparticles was developed for sample preparation in the determination of ciprofloxacin and ofloxacin in meat tissues with the use of capillary electrophoresis. This study is the first utilization of silica-coated magnetic nanoparticles with attached C18 chains to extract fluoroquinolones from meat tissues. This method is therefore characterized by a very simple sample preparation procedure, but on the other hand, by satisfactory precision and accuracy. Magnetic nanoparticles with an appropriately modified surface were placed in an Eppendorf tube, then conditioned with methanol, next rinsed with water and, finally, a homogenized tissue sample was added. At the neutral pH of the sample solution, these compounds do not have a charge and are able to adsorb on the modified particles. After extraction, the nanoparticles were dried and, then, desorption of analytes was conducted with the use of a mixture of 0.1 mol/L HCl and acetonitrile (1:1). This approach made it possible to purify the sample matrix and to obtain satisfactory LOQ levels for the method using the CE technique with UV-Vis detection. In this method, the LOD and LOQ values for both analytes were 0.04 nmol/g tissue and 0.15 nmol/g tissue, respectively. The calibration curves were linear in the entire concentration range, and the accuracy and the recovery of the method were at the satisfactory levels. The square value of the linear correlation coefficients (R^2^) for Cpx and Ofx were 0.9995 and 0.9992, respectively. The precision value of the method was within the range of 3–11% and accuracy was in the range of 93–110%.

## 1. Introduction

**Figure 1:**
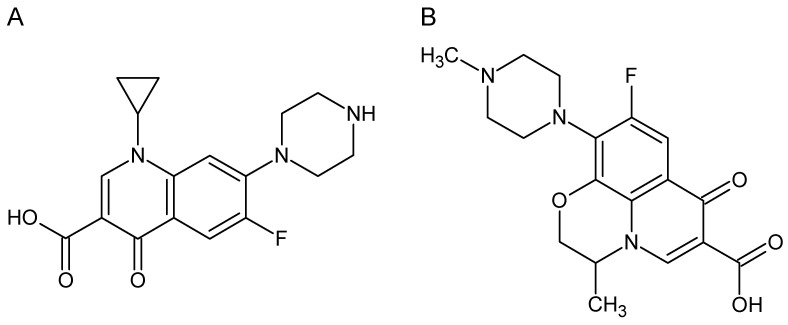
Structures of ciprofloxacin (**A**) and ofloxacin (**B**).

At present, it is difficult to imagine animal husbandry without the prophylactic use of drugs to avoid diseases or as growth promoters, so more and more breeders are choosing to administer drugs to animals, including antibiotics. The growth-intensive method of animal husbandry is forcing breeders to use such drugs for artificial, but rapid, weight gain in animals [1,2,3]. A frequently used group of compounds in veterinary medicine are fluoroquinolones (FQLs), of which ciprofloxacin (Cpx) and ofloxacin (Ofx) are representatives [4]. FQLs exhibit a much broader spectrum of activity against bacteria, but also much better pharmacokinetics compared to first-generation quinolones (a group of antibiotics containing a bicyclic core bound to a 4-quinolone compound); they also exhibit a lower binding to proteins, higher drug tolerance, lower toxicity and longer half-life [5,6]. Cpx (1-cyclopropyl-6-fluoro-4-oxo-7-piperazin-1-ylquinoline-3-carboxylic acid) and Ofx (7-fluoro-2-methyl-6-(4-methylpiperazin-1-yl)-10-oxo-4-oxa-1-azatricyclotrideca-5(13),6,8,11-tetraene-11-carboxylic acid) are quinolone compounds, belonging to the second generation of FQLs, having cyclopropyl, carboxylic group, fluorine atom and piperazin-1-yl in its structure [7], as shown in Figure 1.

FQLs inhibit DNA synthesis by cutting bacterial DNA in DNA gyrase and type IV topoisomerase enzyme complexes, causing rapid bacterial death. They exhibit a broad spectrum of activity against Gram-positive and Gram-negative bacteria [6,8,9].

As a result, they are considered very universal antibiotics, and consequently they are used very often and unfortunately not infrequently overused. This is dangerous due to the fact that the misuse of these drugs can cause public health difficulties, such as allergic reactions and antibiotic resistance. Antibiotic resistance is one of the headline public health problems at present, as bacterial resistance to antibiotics makes it difficult and often impossible to treat bacterial infections. Therefore, the administration of antibiotics to animals should be discontinued for an appropriate time before the animal is slaughtered—the withdrawal time—so the residues of compounds harmful to consumer health are not found in the food. If such a recommendation is not respected, residues of veterinary drugs may remain in the food until the meat is consumed by humans, posing a potential health risk to consumers [10,11].

As a result, laboratories are focusing on developing new, simple but sensitive and precise methods for the determination of hazardous compounds in food [12,13,14]. Several methods for the determination of FQLs in different matrices based on HPLC-UV (in plasma [9,15,16], urine [15,16] and wastewaters [17]) or LC-MS (in animal products [18,19] and wastewaters [8,20]) techniques were developed. The CE technique was also used for the determination of FQLs in water [21] or in water, urine and milk [22]. These methods are significant analytical tools mainly due to their high efficiency and satisfactory sensitivity. Unfortunately, the majority of these methods are based on chromatographic techniques that require the use of large volumes of harmful organic solvents, mainly for the preparation of mobile phases. In addition, methods that utilize MS detection require very expensive and sophisticated equipment, and laboratories often do not possess such apparatus. Considering the above, CE-based methods should gain popularity and be more developed.

The CE technique especially equipped with a UV-Vis detector is an excellent tool for separating compounds that acquire a charge in appropriate environments. Although it is among the universal ones and seems to be an ideal solution, it has some important limitations [23]. Unfortunately, due to the small volume of the sample introduced into the capillaries and the short optical pathway of the detector, this technique has relatively low concentration sensitivity. Therefore, in practice, it is decided to implement an analyte concentration step in the sample preparation procedure or in the capillary directly prior to the analysis. In the present work, a method of concentration with the use of magnetic particles (MPs) of Fe_3_O_4_ coated with silica and modified with C-18 chains is developed. MPs perform a function comparable to the stationary phase in the classical SPE technique. It also turns out that, due to the ease of manipulation of the position of the MPs inside the CE capillary using a magnetic field, such extraction can be successfully conducted during the in-line analysis, thus automating the process and minimizing the number of errors made by the analyst. In the literature, there are several methods describing the application of magnetic SPE using CE [24,25,26,27,28,29,30]. However, there is no method for the determination of FQLs that uses CE combined with magnetic SPE extraction on silica-coated Fe_3_O_4_ particles modified with C-18 chains. Therefore, we decided to develop such a methodology for FQL determination in animal tissues. During the experiments, Cpx and Ofx were used as example compounds representing FQLs.

## 2. Results and Discussion

### 2.1. Sample Preparation

#### 2.1.1. Weight of the Magnetic Nanoparticles

**Figure 2:**
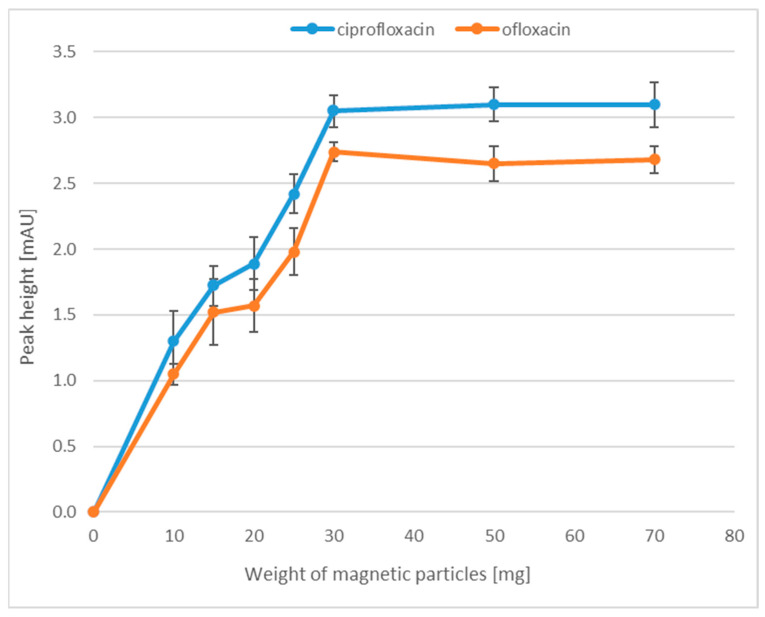
The relationship between the peak height and the weight of MPs.

In the first step of the optimization of the magnetic particle extraction, we focused on the selection of the appropriate MP weight for the extraction. This is a very important step in the selection of sample preparation parameters, because an insufficient number of MPs causes a low extraction efficiency, while too many nanoparticles make it difficult to collect the liquid above them (the turbid solution). We checked what weight of MPs would ensure a high efficiency and at the same time would not cause difficulties during the extraction process. For this purpose, all tested samples were prepared in accordance with Section 3.6, whereas for the extraction of FQLs, the following weight of MPs, i.e., 10, 15, 20, 25, 30, 50 and 70 mg, were tested. Each sample was prepared in three replicates. As shown in Figure 2, the highest efficiency was observed when the weight of nanoparticles was 30 mg. A further increase in MP mass did not increase the extraction efficiency.

#### 2.1.2. Time of Adsorption

**Figure 3:**
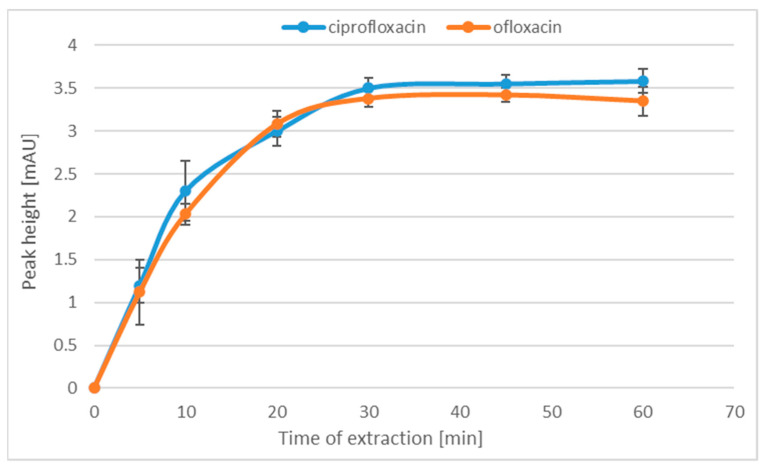
The relationship between the peak height and the time of extraction.

The next extraction-optimized parameter was the adsorption time of the analytes on the MPs. In this step, the influence of the adsorption time on the extraction efficiency was checked. For this purpose, the extraction of MPs was conducted for 5, 10, 20, 30, 45 and 60 min. Each sample was prepared in three replicates. As can be seen in Figure 3, the extraction efficiency increases only up to 30 min and the subsequent extension of the extraction time does not increase the efficiency of the process.

#### 2.1.3. Time of Desorption

**Figure 4:**
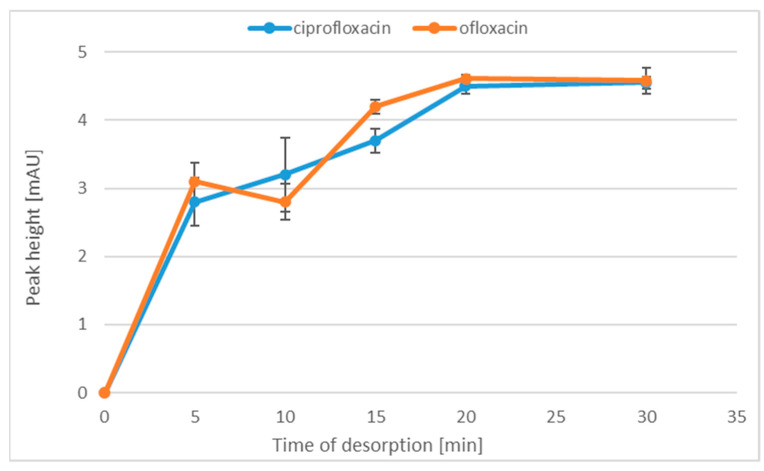
The relationship between the peak height and the time of desorption.

After the optimization of the adsorption time, we decided to check the relation between the extraction efficiency and the analyte desorption time. Therefore, desorption was conducted successively for: 5, 10, 15, 20 and 30 min. Each sample was prepared in triplicate. The results of the experiments are presented in Figure 4. It was found that the extraction yield increased up to the desorption time of 20 min. For longer desorption times, no changes in the height of analytical signals were noted. Therefore, in order not to extend the sample preparation procedure, we chose the MP analyte desorption time of 20 min.

#### 2.1.4. The Volume of the Desorbing Solvent

In the last step of the extraction procedure optimization, the influence of the solvent volume on the analyte desorption efficiency was checked. The following solvent volumes of HCl:ACN (1:1 *v*/*v*) mixture were tested: 200, 400, 600, 800 and 1000 µL. As expected, because a smaller volume of the solvent ensures less dissolution of the sample, the best extraction efficiency was obtained when the analyte was desorbed with 200 µL of the solvent. The reason for testing the volume of the HCl:ACN (1:1 *v*/*v*) mixture within the range of 200–1000 µL is that a volume of desorption solvent lower than 200 µL caused difficulty in sample mixing, while a volume of solvent greater than 1000 µL diluted the sample too much. We also checked whether a better efficiency would be obtained when the analytes were desorbed with a solvent volume of 800 µL and then the sample was evaporated to dryness, and then the residue was dissolved in a smaller (50 µL) volume of solvent. It turned out that such a solution, i.e., introducing the evaporation step into the sample preparation process, is much more advantageous. Therefore, we decided to add this step to the sample preparation procedure.

The main aim of the research was to develop the parameters for the extraction of fluoroquinolones with the use of magnetic iron nanoparticles coated with C-18 modified silica. Very similar results of the experiments in terms of the weight of MPs, sorption and desorption times, and the volume of desorbing solvent were obtained for both analytes.

#### 2.1.5. Sensitivity Enhancement Factor

The sensitivity enhancement factor (*SEF*) for the method was also determined. SEF characterizes the degree of concentration of the analyte with the use of the developed method, where the extraction efficiency is determined by the comparison of the size of the analyte peak obtained using the method with the extraction step and the analyte peak without extraction step. In this work, the following formula was used to calculate the SEF coefficient:

$$SEF=\frac{A_{after\;extraction}}{A_{without\;extraction}}\cdot \frac{C_{without\;extraction}}{C_{after\;extraction}}$$

where $A_{after\;extraction}$—peak area of the analyte for tissue analysis after sample extraction; $A_{without\;extraction}$—peak area of the analyte for tissue analysis without extraction; $C_{after\;extraction}$—concentration of the analyte in the sample, which was analyzed with the extraction step; and $C_{without\;extraction}$—concentration of the analyte in the sample, which was analyzed without the extraction step. The tissue samples were prepared according to the procedure described in the Sample preparation Section. Each sample was prepared in triplicate, and the *SEFs* for Cpx and Ofx calculated from the above equation were 127.4 and 103.7, respectively.

#### 2.1.6. Calibration and Other Validation Data

**Figure 5:**
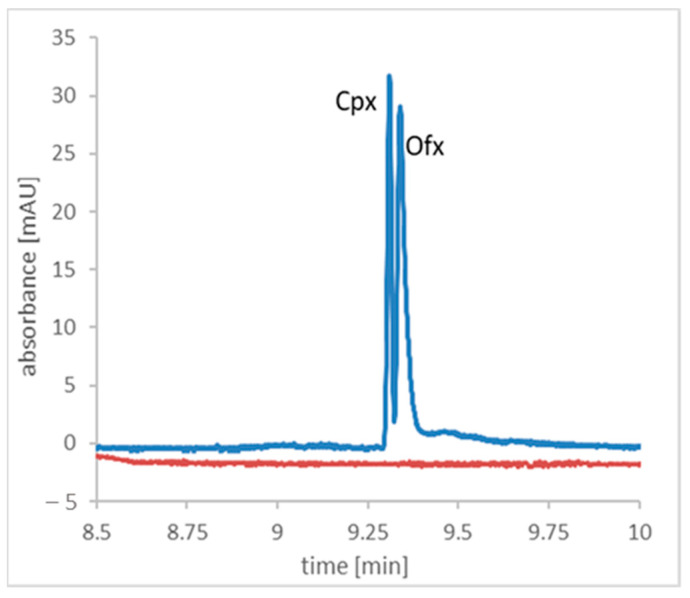
The representative electropherogram of the tissue sample (orange line) and tissue sample spiked with 4 nmol/g ciprofloxacin and ofloxacin (blue line).

The developed procedure was validated in accordance with the criteria defined for the analysis of biological samples [31]. The limit of detection (LOD) and limit of quantification (LOQ) were determined experimentally. The LOD value was the concentration of the analyte for which the signal is three times higher than the baseline noise, and the LOQ was the concentration of the analyte for which the signal was nine times higher than the baseline noise (0.2 mAU). In this method, the LOD and LOQ values for both analytes were 0.04 nmol/g tissue and 0.15 nmol/g tissue (5 ng/mL for Cpx and 5.4 ng/mL for Ofx), respectively. The LOD and LOQ values for our procedure were higher than for the methods that use MS detection [1]: the LOD value for Cpx and Ofx was 0.005 ng/mL and the LOQ value was 0.015 ng/mL for both analytes [32]; the LOD values for Cpx and Ofx were 0.15 ng/L and 3.0 ng/L, respectively; the LOQ for Cpx and Ofx was 0.2 ng/L and 10 ng/L, respectively [33]; the LOD value for Cpx and Ofx was 10.1 ng/L and 3.1 ng/L, respectively; the values of LOQ for Cpx and Ofx were 33.8 and 10.4 ng/L, respectively. However, they were lower than for the CIEF method [34] (LOD value for Cpx was 0.34 µg/mL and for Ofx was 1.20 µg/mL) and for other CE procedures [35] (for Cpx, the LOQ value was 0.0125 mg/mL). In turn, the LOQ value of our proposed methodology was similar to that of another CE method [36], where the LOQ for Cpx was 0.3 nmol/g tissue and for Ofx was 0.25 nmol/g tissue. The total analysis time for our method was 77 min. This time is similar to that of the LLL-SDME-CE procedure [23] (61 min), but shorter than that of the EEM-ANWE approach [4] (155 min) and longer than that of the CE procedure [36] (32 min). Five-point calibration curves for both Cpx and Ofx were constructed in the concentration range from 2 to 10 nmol/g tissue. Each series was repeated three times. The resulting calibration curves (Figure 5) were linear in the concentration range tested. The square value of the linear correlation coefficients (R^2^) for Cpx and Ofx were 0.9995 and 0.9992, respectively. The calculated equations of the calibration curves were as follows: y = (23.648 ± 0.310)x + (0.465 ± 0.026) for Cpx and y = (16.021 ± 0.263)x + (0.715 ± 0.017) for Ofx. The calibration data are summarized in Table 1.

The precision (expressed as *RSD*) of the points of the calibration curve for Cpx ranged from 3.1% to 9.3%, and that of the points of the calibration curve for Ofx ranged from 0.5% to 7.9%, while the accuracy (expressed as recovery) for Cpx ranged from 94.6% to 99.5% and for Ofx from 90.1% to 95.7%. *RSD* was calculated based on the formula shown below:

$$RSD\left[\%\right]=\frac{SD\cdot 100\%}{x}$$

where *x* is the average value of the concentration and *SD* is the standard deviation of *x*.

The accuracy was calculated by the formula shown below:

accuracy [%]=100%−Error Rate

where $Error\;Rate=\frac{\left|Observed\;Value-Actual\;Value\right|}{Actual\;Value}$ · 100%

These values are in accordance with the criteria for the analysis of biological samples [31]. To test the intra-day and inter-day precisions of the method, three concentrations were selected from a range of the calibration curve, and the samples were prepared and analyzed. We chose the first concentration (3 nmol/g tissue) from the beginning of the range of the calibration curve, the next concentration (5 nmol/g tissue) represented the middle of the range of the calibration curve, and the final concentration was at the end of the range of the calibration curve (9 nmol/g tissue). Both the precision (RSD of 3–11%) and accuracy (in the range of 93–110%) of the method were at a satisfactory level according to the criteria for the analysis of biological samples [31]. After the analysis of the calibration samples, the peak areas of Cpx and Ofx were plotted against the corresponding concentrations of the analyte, and then the calibration curves were fitted using a least-squares linear regression analysis. In the essence of this method, the equation of the calibration curve is a certain averaging of the results of the dependence of the peak area on the analyte concentration. Therefore, the points of the calibration curve may lie above the averaged curve—then, the recovery is above 100%. Another reason may be the result of the errors made by the analyst. All validation data are shown in Table 2.

The validated method was utilized to analyze a real meat tissue samples. For this purpose, different types of meat tissues were analyzed. It was found that there is no matrix influence in what is shown in the representative electropherogram obtained after the analysis of the chicken liver tissue and spiked chicken liver tissue (Figure 5). This type of tissue was chosen because FQLs are cumulated in the liver. Figure 5 shows that the peaks are not well-separated to the baseline, but this was not the aim of the work. The main aim of the work was to develop an MP extraction procedure and optimize its parameters. The electrophoretic conditions were taken from our previous work [37]. In addition, the signals of Cpx and Ofx was recorded at two different wavelengths, which greatly facilitated the integration of the peaks. Nevertheless, the validation parameters are in accordance with the criteria, which nevertheless makes the method reliable.

## 3. Materials and Methods

### 3.1. Instruments

For all experiments, an Agilent 7100 CE System (Waldbronn, Germany) coupled with UV-Vis absorbance diode-array detector and equipped with automatic injector was used. The bare fused silica capillary (Polymicro Technologies, Phoenix, AZ, USA) with a total length of 40 cm (effective length of 31.5 cm) and an inner diameter of 75 µm was used as the separation column. To measure migration times, peak heights, peak areas, and other data Agilent ChemStation Rev. B.04.02. SP1 software were used. The Millipore Milli-Q-RG System (Waterford, Ireland) was used for the deionization of water. A pH meter (Mettler-Toledo, Columbus, Ohio, USA) was used to adjust the pH of the buffer solutions and to shake the samples vortex. The Labconco CentriVap (Kansas City, MO, USA) was used to lyophilize samples, and a centrifuge with a fast cool function (Mikro 220R, Hettich Zentrifugen, Tuttlingen, Germany) was used to centrifuge the samples. Fourier-transform infrared spectroscopy (FTIR) analysis was performed on a Nicolet iS50FT-IR spectrometer (Thermo Scientific, Madison, WI, USA) with a DTGS detector and the EasyDiff (PIKE Technologies, Fitchburg, WI, USA) diffuse reflectance accessory over the spectral region from 4000 to 400 cm^−1^, using 64 sample scans and a resolution of 4 cm^−1^.

### 3.2. Chemicals

Sodium hydroxide (NaOH) was purchased from POCH (Gliwice, Poland); sodium phosphate dibasic (Na_2_HPO_4_), sodium dihydrogen phosphate (NaH₂PO₄), trisodium phosphate (Na_3_PO_4_), N,N-dimethylformamide, ammonium acetate and acetonitrile were purchased from Sigma (Steinheim, Germany); and methanol (CH_3_OH) was obtained from J.T. Baker (Deventer, Netherlands). The standard of the analytes Cpx (C_17_H_18_FN_3_O_3_) and Ofx (C_18_H_20_FN_3_O_4_), iron (II,III) oxide (size of 50–100 nm) and toluene were obtained from Sigma Aldrich (Saint Louis, MO, United States of America). Octadecyltrimethoxysilane was obtained from Thermo Scientific (Shanghai, China). The buffer pH was adjusted by potentiometric titration.

### 3.3. Preparation of the Magnetic Particles

The preparation of MPs was conducted according to the procedure of Baciu et al. [38]. Briefly, 1 g of Fe_3_O_4_ MPs (average diameter of 75 nm) was dispersed in 100 mL of isopropanol/deionized water (90:10, *v*/*v*) under N_2_ flow with constant stirring. Subsequently, the pH of the solution was adjusted to 9 with 1.5 mol/L ammonia solution, and 5 mL of tetraethyl orthosilicate was added dropwise.

The mixture was stirred at room temperature for 16 h and then heated to 50 °C for 8 h. The silica-coated MPs were sequentially washed with deionized water and isopropanol, and then vacuum-dried (24 °C, 500 Pa) for 24 h. In the next step, the surface modification of silica-coated magnetic particles with the use of C18 chains was conducted. For this purpose, the obtained silica-coated MPs (0.5 g) were dispersed in 100 mL of dimethylformamide/toluene (80:20, *v*/*v*) under a N_2_ flow with constant stirring. Then, 10 mL of trimethoxyoctadecylsilane was added dropwise, and the solution was stirred for 24 h at room temperature. Finally, the finished functionalized magnetic particles were washed three times with toluene and then dried under vacuum (24 °C, 500 Pa) overnight.

**Figure 6:**
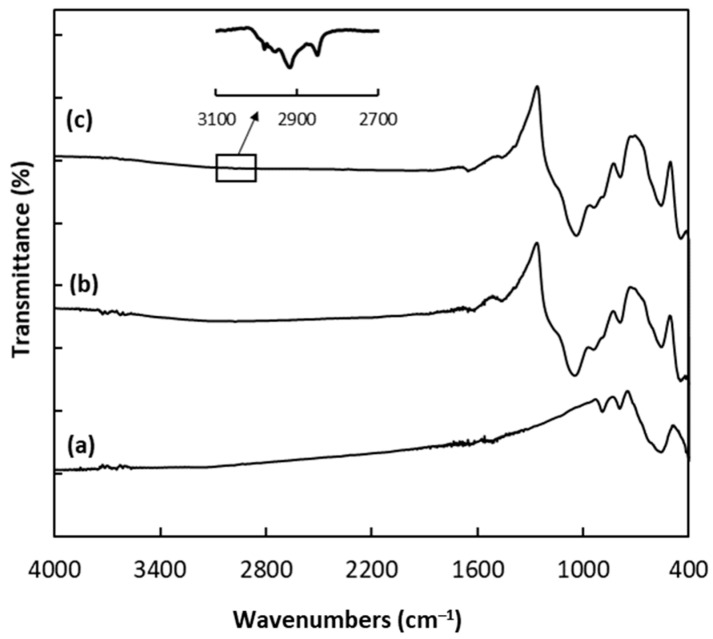
FTIR spectra of (**a**) Fe_3_O_4_ MPs, (**b**) Fe_3_O_4_ coated with silica and (**c**) Fe_3_O_4_ coated with silica and C18.

The FTIR spectra of the MPs at each stage of the synthesis were compared as shown in Figure 6. A characteristic band in the FTIR spectrum of MPs (Figure 6a) appears at 562 cm^−1^ and was assigned to the Fe–O–Fe vibration in magnetite nanoparticles [38,39]. After the modification of the magnetic nanoparticles with the silica coating, two adsorption bands with the maximum in 1038 cm^−1^ and 785 cm^−1^ were observed, resulting from the asymmetric and the symmetric stretching vibrations of Si–O–Si groups [38,39]. Moreover, the peak at ~930 cm^−1^ can be attributed to Si−OH stretching vibrations [38,39], while the band at 445 cm^−1^ can be associated with Si–O rocking vibrations [40]. In addition, the peak at ~1620 cm^−1^ comes from the O–H bending vibration of adsorbed water [38]. The introduction of C18 onto the surface of the silica coating resulted in the appearance of two bands (Figure 6c inset), which correspond to the asymmetric (2915 cm^−1^) and the symmetric (2847 cm^−1^) stretching vibration of methylene groups in the -(CH_2_)_17_CH_3_ chain [41]. Thus, the FTIR spectra confirm the modification of MPs with a silica coating and successful functionalization using C18.

### 3.4. Capillary Preconditioning

The new capillary was conditioned by flushing with a 1 mol/L NaOH solution for 20 min, 0.1 mol/L solution of NaOH for 20 min, then 2 min with deionized water and with BGE for 30 min. At every next day of the work, the capillary was flushed with 0.1 M NaOH solution for 20 min, deionized water for 2 min and 30 min with BGE. At the end of each day, the capillary was flushed with water for 20 min, and the capillary ends were left in the water overnight.

### 3.5. Electrophoretic Conditions

During electrophoretic analyses, 0.1 mol/L phosphate-borate buffer with a pH of 8.40 was used as the BGE. The voltage of 16 kV was applied for the separation, and the temperature of the capillary was 23 °C. The hydrodynamic introduction of the sample solution was conducted at 25 mbar for 10 s. The UV-Vis detection of Cpx and Ofx was performed at the analytical wavelengths of 271 nm and 285 nm, respectively.

### 3.6. Sample Preparation

The purchased animal tissues (liver and kidneys) from local stores were divided into smaller portions and stored at a reduced temperature (−20 °C). The homogenization parameters of the animal tissues were adapted from our previous method [41]. To homogenize the sample, 0.2 g of tissue weighed and then placed in a 3 mL polypropylene tube with 2 mL of 0.2 mol/L phosphate buffer (pH 7.00) and homogenized to allow for further sample preparation. This mixture was centrifuged at 13,680× *g* (12,000 rpm) for 10 min, and the supernatant was used for the subsequent procedure. For each sample, 30 mg of MPs were weighed and placed in a polypropylene tube. Then, 1.5 mL of methanol was added to condition the surface of the MPs and vortexed for 5 min. In the next step, methanol was removed, and 1.5 mL of H_2_O was added, vortexed for 2 min, and then, after water removal, 1.5 mL of the sample solution (supernatant) was added. The adsorption of analytes was conducted for 30 min; then, the sample solution was removed, and the MPs were dried. Subsequently, in order to induce the desorption of the analytes, 800 µL of the 0.1 mol/L HCl:ACN (1:1 *v*/*v*) mixture was added to the MPs and vortexed for 20 min. A sample was then collected in a polypropylene tube and lyophilized to dryness. Next, the residue was dissolved in 50 µL of 0.002 mol/L HCl and subjected to electrophoretic analysis. To dissolve the evaporation residue, we used a low-concentration HCl to reduce the conductivity of the sample, which significantly improved CE separation.

### 3.7. Method Validation

After optimizing all sample preparation parameters, the method was validated. We decided to validate the method for two representatives of FQLs in order to prove that magnetic extraction can be performed for several antibiotics from this group simultaneously. Limit of detection (LOD), limit of quantification (LOQ), method precision and accuracy were determined in accordance with the Food and Drug Administration (FDA) Criteria for Analytical Procedures and Method Validation [31].

### 3.8. Calibration of the Method

Stock solutions of each analyte, which were then used to prepare the calibration solutions, were prepared by the appropriate dilutions of the standard solution of the analytes in 0.1 mol/L HCl. We prepared three series of Cpx and Ofx solutions with concentrations in the range of 2–10 nmol/g tissue for calibration. These solutions were prepared according to the following procedure: the animal tissue was homogenized with 0.2 mol/L, pH 7.00 and 1:10 phosphate buffer (g/mL) in a 3 mL polypropylene tube. The homogenate was then centrifuged at 13,680× *g* (12,000 rpm) for 10 min. Subsequently, increasing amounts of the standard working solutions were added to the supernatant to obtain the following concentrations for both Cpx and Ofx: 2 nmol/g tissue, 4 nmol/g tissue, 6 nmol/g tissue, 8 nmol/g tissue and 10 nmol/g tissue. The calibration solutions were extracted according to the following procedure: A total of 30 mg of MPs were placed in a polypropylene tube. Then, 1.5 mL of methanol was added to condition the bed and vortexed for 5 min. In the next step, methanol was removed, and 1.5 mL of H_2_O was added and vortexed for 2 min; then, after the removal of water, 1.5 mL of the sample solution of the appropriate concentration was added. The extraction was conducted for 30 min; then, the aqueous phase of the sample was removed, and the nanoparticles were dried. Subsequently, in order to induce the desorption of the analytes, 800 µL of the mixture of 0.1 mol/L HCl:ACN (1:1 *v*/*v*) was applied to the MPs and vortexed for 20 min. Next, the sample was lyophilized to dryness, dissolved in 50 µL 0.002 mol/L HCl and subjected to electrophoretic analysis. After the analysis, the peak areas of Cpx and Ofx were plotted against the corresponding concentrations of the analyte, and then the calibration curves were fitted using a least-squares linear regression analysis.

## 4. Conclusions

A new, fast and simple extraction procedure was developed that can be used for real sample preparation in the CE determination of Cpx and Ofx. The described procedure is characterized by a very simple and relatively quick extraction and allows obtaining results at a satisfactory level. In this study, Fe_3_O_4_ nanoparticles coated with C-18 silica were selected as the stationary phase for the magnetic extraction of certain FQLs. The described procedure is very easy and does not require complicated equipment while maintaining a relatively high sensitivity—a plastic test tube and appropriate magnetic nanoparticles are enough. The developed procedure does not use significant large volumes of organic solvents. In our method, the consumption of solvents per sample is 1.525 mL. The usage of organic solvents in the presented methodology is much smaller than that in CE [36] (10 mL per sample) and UHPLC [2] (5.84 mL per sample) procedures, but larger than that in the LLL-SDME procedure [23] (350 µL). In addition, the developed method is sensitive and precise, and it is characterized by a good linearity and accuracy. In this method, the LOD and LOQ values for both analytes were 0.04 nmol/g tissue and 0.15 nmol/g tissue, respectively. The square values of the linear correlation coefficients (R^2^) for Cpx and Ofx were 0.9995 and 0.9992, respectively. The precision value of the method was within the range of 3–11% and accuracy was in the range of 93–110%. The maximum residue limit for the tested antibiotics is 200 µg/kg tissue [42] (0.6 nmol/g tissue for Cpx and 0.55 nmol/g tissue for Ofx). It follows that it is possible to detect and quantify analytes with such content in meat with the use of the developed procedure. The SEFs for Cpx and Ofx calculated from the above equation were 127.4 and 103.7, respectively. The total analysis time, which includes sample preparation and electrophoretic analysis, of our method is 77 min. The time is similar to that of the LLL-SDME-CE procedure [23] (61 min), but shorter than that of the EEM-ANWE approach [4] (155 min) and longer than that of the CE procedure [36] (32 min).

The described work presented the use of silica-coated magnetic nanoparticles with attached C18 chains for the extraction of fluoroquinolones in meat tissues for the first time. The use of magnetic nanoparticles modified with the use of the described method seems to be an ideal application because of the physicochemical properties of fluoroquinolones. With the appropriate manipulation of the pH of the sample solution, these compounds do not have a charge and are able to adsorb on the modified particles. This approach enables the concentration of the analyte in the sample, but also the purification of the sample matrix. However, the concept for such an analytical procedure requires further development in order to use this type of extraction, e.g., inside the CE capillary, which would simplify sample preparation procedure and probably significantly improve SEF coefficients. Furthermore, better results can be achieved by using a properly selected internal standard in terms of the precision of the method. We believe that subsequent research in this direction is needed, as this approach seems to be an excellent, simple and cheap tool that will be useful in the analysis of meat in terms of its antibiotic content.

Considering all the advantages of our method, we believe that this type of extraction can be successfully used in the analysis of real samples for Cpx and Ofx content, which encourages further research in this area.

## Figures and Tables

**Table 1:** Analytical data.

LOD	LOQ	Calibration Concentration Range	Equations of the Calibration Curve	R^2^
Ciprofloxacin				
0.04 nmol/g tissue	0.15 nmol/g tissue	2 to 10 nmol/g tissue	y = (23.648 ± 0.310)x + (0.465 ± 0.026)	0.9995
Ofloxacin				
0.04 nmol/g tissue	0.15 nmol/g tissue	2 to 10 nmol/g tissue	y = (16.021 ± 0.263)x + (0.715 ± 0.017)	0.9992

**Table 2:** Validation data—results.

Added * (nmol/g Tissue)	Intra-Day				Inter-Day			
	Found ± SD (nmol/g Tissue)	Confidence Interval (nmol/g Tissue	RSD (%)	Accuracy (%)	Found ± SD (nmol/g Tissue)	Confidence Interval (nmol/g Tissue	RSD (%)	Accuracy (%)
Ciprofloxacin								
3.0	3.1 ± 0.2	3.1 ± 0.4	6.4	97.9	3.0 ± 0.2	3.0 ± 0.4	5.7	98.8
5.0	5.0 ± 0.2	5.0 ± 0.5	3.2	99.5	5.3 ± 0.4	5.3 ± 0.9	7.0	94.6
9.0	8.9 ± 1.0	8.9 ± 2.5	11.2	98.4	9.3 ± 0.3	9.3 ± 0.8	3.7	96.6
Ofloxacin								
3.0	3.0 ± 0.1	3.0 ± 0.3	3.7	95.7	2.8 ± 0.1	2.8 ± 0.1	2.1	94.6
5.0	5.3 ± 0.4	5.3 ± 0.9	7.8	93.0	4.7 ± 0.4	4.7 ± 0.9	7.7	93.0
9.0	9.3 ± 0.9	9.3 ± 2.2	9.3	94.0	9.9 ± 0.3	9.9 ± 0.7	3.0	90.1

* *n* = 3.

## Data Availability

CE data are available from the authors.

## Sample Availability

Meat tissue samples are not available from the authors.
